# BMP5 signalling in beta cells and the impact on insulin secretion in the context of type 2 diabetes

**DOI:** 10.1007/s00125-025-06457-9

**Published:** 2025-06-05

**Authors:** Esmée Dekker, Twan J. J. de Winter, Amadeo Muñoz Garcia, Natascha de Graaf, Maaike J. Roodzant, Eelco J. P. de Koning, Françoise Carlotti

**Affiliations:** Department of Internal Medicine, Leiden University Medical Center (LUMC), Leiden, the Netherlands

**Keywords:** Beta cell identity, Beta cells, BMP5, Diabetes mellitus, Insulin secretion, Mitochondrial metabolism

## Abstract

**Aims/hypothesis:**

Identifying signalling pathways that are important in pancreatic beta cell stress and failure can give insight into possible treatment options to prevent the loss of functional beta cell mass in diabetes. The bone morphogenetic protein (BMP)/SMAD family member *BMP5* has been reported to be specifically expressed in human beta cells, but its function is unknown. Here we hypothesised that BMP5 plays a role in the maintenance of beta cell function.

**Methods:**

We assessed the expression of *BMP5* in publicly available single-cell RNA sequencing (scRNA-seq) datasets of primary human pancreatic islet cells from donors with or without type 2 diabetes, or islets exposed to stress conditions. Human islets and EndoC-βH1 cells were exposed to recombinant BMP5 and used for gene analysis, mitochondrial respiration and glucose-stimulated insulin secretion tests. In addition, we performed lentivirus‐mediated knockdown using short hairpin RNAs targeting *BMP5* in human islets and EndoC-βH1 cells for gene expression and glucose-stimulated insulin secretion analyses.

**Results:**

scRNA-seq data revealed that *BMP5* is the most predominantly expressed BMP ligand in beta cells. *BMP5* and its target genes are upregulated in beta cells from donors with type 2 diabetes. Enhanced BMP5 signalling triggered an upregulation of stress-related genes, and a reduction in glucose-stimulated mitochondrial oxygen consumption and insulin secretion. In contrast, downregulation of *BMP5* in primary human islets enhanced beta cell function, which was associated with increased expression of key beta cell genes.

**Conclusions/interpretation:**

Altogether, these findings point toward a role for BMP5 in the regulation of beta cell function.

**Graphical Abstract:**

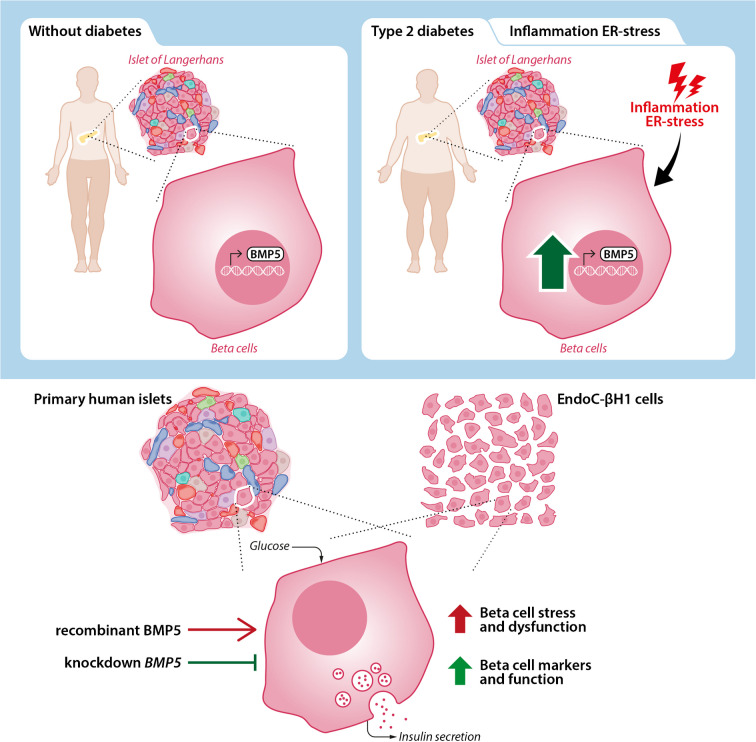

**Supplementary Information:**

The online version contains peer-reviewed but unedited supplementary material available at 10.1007/s00125-025-06457-9.


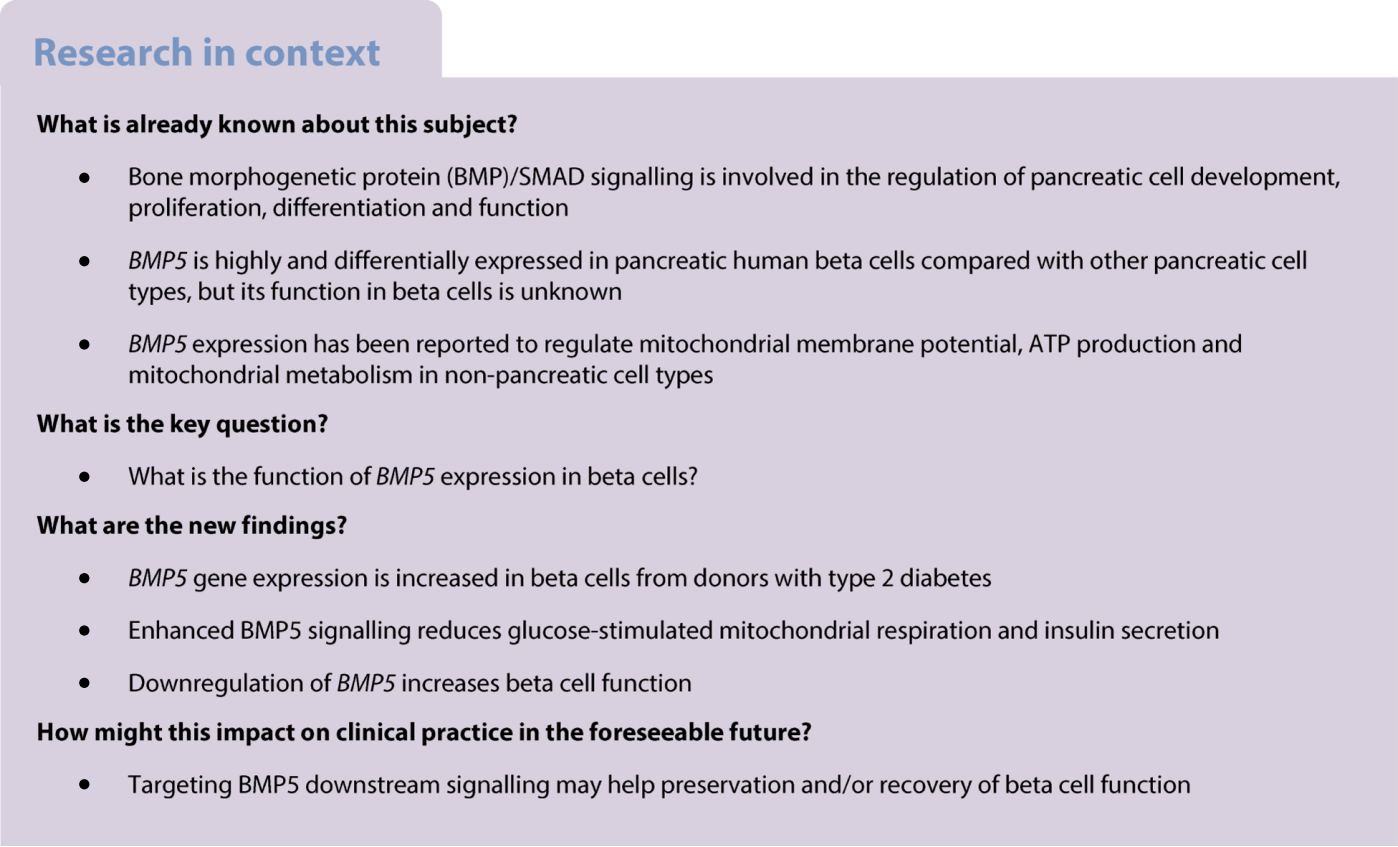


## Introduction

The pathogenesis of type 2 diabetes mellitus arises from inadequate insulin secretion caused by beta cell dysfunction, which often occurs alongside the onset of insulin resistance [1], ultimately causing the beta cells to be unable to compensate for the high insulin demand. The inflammation process during the onset of diabetes has been extensively studied as a key mechanism leading to beta cell failure and beta cell death [2–9]. Various studies have described islet infiltration by macrophages and elevated levels of cytokines, including IL-1β, in type 2 diabetes [10–14]. The proinflammatory cytokines IL-1β and IFNγ have been shown to trigger cell death through ER stress activation by the pro-apoptotic mediators activating transcription factor 3 (ATF3) and C/EBP homologous protein (CHOP) [15, 16]. The initial response of beta cells during the onset of type 2 diabetes is to adapt their insulin secretion to maintain euglycaemia and to compensate for peripheral insulin resistance [17–20]. Although the functional adaptation of beta cells has been described, the primary molecular mechanisms are yet to be determined. Studying the underlying mechanisms of functional adaptation and inflammation will provide insight into means to prevent the loss of beta cell mass and restore beta cell function in diabetes.

Bone morphogenetic proteins (BMPs) are a group of growth factors that belong to the TGFβ superfamily. BMP ligands act through binding transmembrane receptor serine/threonine kinases, and are classified as BMP type 1 and type 2 receptors. In the canonical signalling pathway, activated receptors phosphorylate and thereby activate SMAD-1/5/9, which associate with SMAD4, mediating BMP signalling. Various non-canonical pathways have been described as being activated by BMPs, such as the phosphoinositide 3-kinase, extracellular signal-regulated kinase and P38 pathways [21]. BMPs have widespread functions and pleiotropic, context-dependent consequences.

*BMP5* has been reported to be specifically expressed in beta cells in the adult pancreas [22–25], and BMP5 has been shown to promote fetal pancreatic epithelium formation during development [26]. However, the function of BMP5 in adult beta cells is unknown. BMP5 has been suggested to affect mitochondrial function in adipocytes and Schwann cells [27, 28]. Associations have been found between BMP5 and glycerolipid metabolism, mitochondrial membrane potential and ATP production. This implies a potential role for BMP5 in regulating mitochondrial and metabolic activity in beta cells.

In this study, we investigated the role of BMP5 in pancreatic beta cells and its effect on cell identity and function.

## Methods

### scRNA-seq

**Fig. 1:**
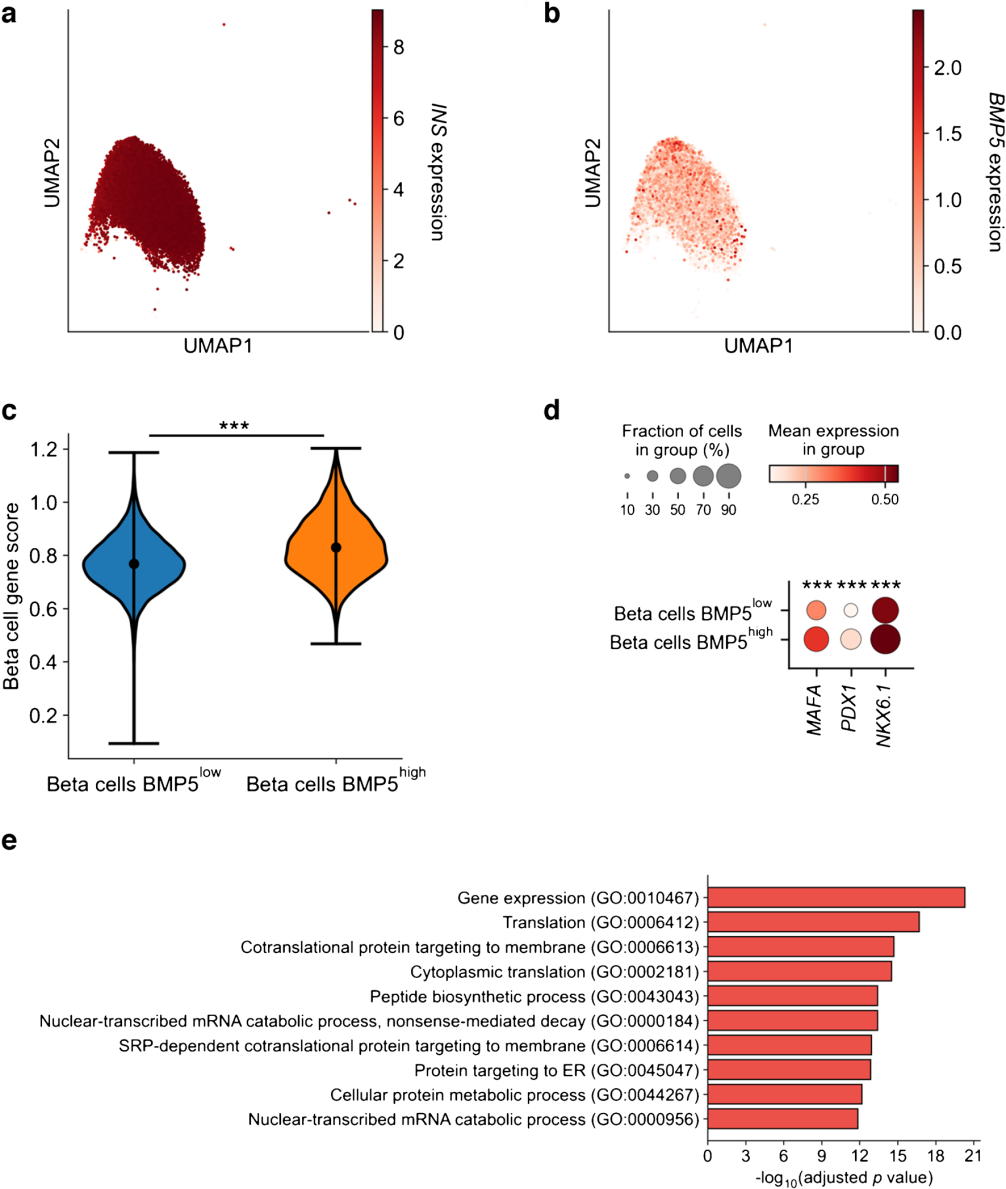
(**a**, **b**) Uniform manifold approximation and projection (UMAP) visualisation of beta cells derived from donors without diabetes through gene expression of (**a**) the canonical identity marker *INS* and (**b**) *BMP5*, after scRNA-seq. (**c**) Violin plot representing the beta cell gene score [31] of beta cells expressing low levels of *BMP5* (BMP5^low^) compared with beta cells expressing high levels of *BMP5* (BMP5^high^). (**d**) Dot plot of *MAFA*, *PDX1* and *NKX6.1* expression in beta cells with low expression of *BMP5* (BMP5^low^) compared with beta cells expressing high levels of *BMP5* (BMP5^high^). (**e**) GO analysis of upregulated genes in beta cells expressing high levels of *BMP5* compared with beta cells expressing low levels of *BMP5*. SRP, signal-recognition particle. The Wilcoxon rank sum test was used to assess statistical significance: ***p<0.001

In the first set of experiments, we used a publicly available single-cell RNA sequencing (scRNA-seq) dataset of human pancreatic islets from donors with type 2 diabetes (*n*=17) or without type 2 diabetes (*n*=27) from the Human Pancreas Analysis Program [29] and imported the curated h5ad object in Python (version 3.8.5). We excluded donors with type 1 diabetes or autoantibodies. We used the cell type annotation described previously [29], and only selected the beta cells for our analysis. Differential gene expression analysis was performed using the Python package ‘Scanpy’ (version 1.9.3) function ‘rank_genes_groups’ with the Wilcoxon rank sum test. Heterogeneity of gene expression was quantified by the CV as previously reported [30], and was calculated as the SD divided by the mean multiplied by 100 using the Python package ‘statistics’. Beta cell gene scores were computed using the Scanpy function ‘score_genes’ in Python, with signature genes retrieved from the Gene Set Enrichment Analysis (GSEA) database (www.gsea-msigdb.org/gsea/index.jsp) [31–33]. BMP5^high^ beta cells were defined as those with a normalised expression level of *BMP5* above zero, compared with the BMP5^low^ cells, in which the normalised expression level of *BMP5* is zero (see electronic supplementary material [ESM] Fig. 1a). Due to the limited sensitivity of single-cell transcriptomics technology to detect low-abundance RNAs, we cannot exclude the presence of low *BMP5* expression in BMP5^low^ cells, which is why use of BMP5^low^ cells was preferred to using BMP5^neg^ cells (those that do not express *BMP5*). Finally, GO term pathway analysis was performed using the Python package ‘GSEApy’ (version 0.10.8).

In a second set of experiments, we used an scRNA-seq dataset (GEO accession number GSE218316) from our lab, generated from isolated primary human pancreatic islets (*n*=3) that were untreated or treated with beta cell stressors. The stressors used were IL-1β (1 ng/ml; R&D Systems), IFNγ (50 ng/ml, R&D Systems) and thapsigargin (0.1 μmol/l, Bio-Connect, Huissen, the Netherlands). The dataset was processed and analysed as described previously [34]. The various treatment conditions were compared using a Wilcoxon rank sum test.

### Cell culture

Human islets were isolated from pancreases of cadaveric donors obtained through the Eurotransplant multiorgan donation programme. Islets were only used for research if they could not be used for clinical purposes and if research consent was present, according to Dutch national laws. Isolation of primary human islets was performed as previously described [35]. Primary human islets were cultured in CMRL 1066 (Corning: supplemented with 17.5 μg/ml ciproxin, 1.05 mg/ml nicotinamide, 43.9 μg/ml gentamicin, 1.75 mmol/l l-glutamine, 8.77 mmol/l HEPES, 9.21% FCS) in ultra-low adherent plates (Corning). The EndoC-βH1 cells were obtained from Univercell Biosolutions (Toulouse, France) [36]. EndoC-βH1 cells were cultured in DMEM (Thermo Fisher Scientific) with 5.5 mmol/l glucose, 2% albumin, 10 mmol/l nicotinamide, 5.5 g/ml transferrin, 6.7 ng/ml selenite, 2% penicillin/streptomycin mix and 50 μmol/l β-mercaptoethanol on pre-coated TPP plates (Sigma-Aldrich). The coating medium consisted of DMEM with 24.75 mmol/l glucose, 1% extracellular matrix gel, 0.2% fibronectin and 1% penicillin/streptomycin mix. The EndoC-βH1 cells were regularly tested for potential mycoplasma infection. The number of different islet donors or cell passages used per experiment is indicated in the figure legends. Information on donor characteristics for the primary human islets derived from donors without diabetes and used in this study is provided in ESM Table 1 (*n*=25).

### In vitro treatments

Primary human islets and cells were treated with IL-1β (1 ng/ml; R&D Systems), IFNγ (50 ng/ml; R&D Systems), thapsigargin (1μmol/l; Bio-Connect, Huissen, the Netherlands) or BMP5 (50 ng/ml; R&D Systems). The duration of the treatments was between 5 and 72 h, as specified in the figure legends. The cells were incubated at 37°C in 5% CO_2_ in a humidified atmosphere.

### Lentivirus-mediated knockdown

Short hairpin RNA (shRNA) lentiviral constructs against *BMP5* or non-target short hairpin control were acquired from the MISSION shRNA library (clones TRCN0000058269 [*BMP5* kd1], TRCN0000058270 [*BMP5* kd2] and non-target control SHC-002, all Sigma-Aldrich) and produced as described previously [37]. Overnight infection of dispersed primary human islets and EndoC-βH1 cells was carried out at a multiplicity of infection of 2 in CMRL/polybrene (8 μg/ml) and DMEM/polybrene (8 μg/ml) for islets and EndoC-βH1 cells, respectively. After approximately 16 h, cells were washed with PBS and cultured in the corresponding medium for 72 h. *BMP5* knockdown experiments were included if *BMP5* mRNA levels in the cells treated with the knockdown constructs were lower compared to the short hairpin control-treated cells.

### RNA isolation and real-time PCR

RNA isolation of cell lysates was performed using RNeasy micro kits (Qiagen). M-MLV reverse transcriptase (Invitrogen), oligo(dT)s (Qiagen), dNTP (Promega), DTT (Invitrogen) and RNAse-OUT (Thermo Fisher Scientific) were used for reverse transcription of the isolated RNA. Quantitative PCR was performed using 1.25 ng cDNA, SYBR Green PCR Master (Bio-Rad) and 10 µmol/l primer mix (forward and reverse) in a Bio-Rad CFX384 Touch real-time PCR detection system. ESM Table 2 lists the primers used.

### Seahorse assay

A seahorse XF cell-culture plate (96 wells) was pre-coated with Matrigel (Corning) and used to culture primary human islets treated with BMP5 or not treated overnight. Mitochondrial function was assessed using an Agilent Seahorse XF Cell Mito stress test kit according to the manufacturer’s instructions. Islets were incubated for 2 h in Krebs buffer (114 mmol/l NaCl, 4.7 mmol/l KCl, 1.2 mmol/l KH_2_PO_4_, 1.16 mmol/l MgSO_4_, 2.5 mmol/l CaCl_2_, 20 mmol/l HEPES, 2.2 mmol/l glucose in MilliQ water [Millipore] with 0.2% BSA). During the assay, glucose, oligomycin, carbonyl cyanide-*p*-trifluoromethoxyphenylhydrazone and rotenone/antimycin A (final concentrations per well of 20 mmol/l, 5 µmol/l, 4 µmol/l and 1 µmol/l, respectively) were sequentially added to the cells. The oxygen consumption rate (OCR) was analysed using an Agilent Seahorse XF Pro analyser.

### Glucose-stimulated insulin secretion

Approximately 50 islet equivalents for each experimental condition were placed in a chamber of a Biorep perifusion system V5. Islets were perfused for 75 min (flow rate 50 μl/min) with low-glucose (2 mmol/l) Krebs buffer (115 mmol/l NaCl, 5 mmol/l KCl, 24 mmol/l NaHCO_3_, 2.2 mmol/l CaCl_2_, 1 mmol/l MgCl_2_, 20 mmol/l HEPES and 0.2% human serum albumin) at pH 7.4. Islets were subsequently perfused with high-glucose Krebs buffer (20 mmol/l; 40 min), low-glucose Krebs buffer (2 mmol/l; 15 min), KCl Krebs buffer (60 mmol/l; 5 min) and finally low-glucose Krebs buffer again (15 min). A human insulin ELISA kit (Mercodia) was used to measure insulin secretion.

### Fluorescein diacetate/propidium iodide staining

Cultured primary human islets that were untreated, treated for 72 h with BMP5 (50 ng/ml) or treated for 5 min with 2% Triton (positive control for cell death) were stained with fluorescein diacetate (10 µg/ml, Thermo Fisher Scientific) and propidium iodide (20 µg/ml, Thermo Fisher Scientific) in PBS protected from light. The cells were imaged using an EVOS M7000 imaging system (Thermo Fisher Scientific).

### Western blot

Primary human islet cells were washed with PBS and lysed using RIPA lysis and extraction buffer with 1/100 protease and phosphatase inhibitor (Thermo Fisher Scientific). The lysates were passed through a 26-gauge needle. The protein concentration was measured using a BCA protein content kit (Thermo Fisher Scientific). Aliquots of protein (10 μg) were loaded onto a 12% Mini-PROTEAN TGX stain-free protein gel (Bio-Rad) and subsequently transferred to 0.2 µmol/l PVDF membranes (Trans-blot turbo mini 0.2 µmol/l PVDF transfer packs, Bio-Rad). Membranes were blocked for 1 h with 5% milk in PBS-Tween, then incubated with the primary antibody for 1 h at room temperature or overnight at 4°C. After washing with PBS-Tween, the secondary antibody was applied to the membrane and incubated at room temperature for 1 h. The Supersignal West Pico PLUS chemiluminescent substrate (Thermo Fisher Scientific) was used to visualise the blots on a ChemiDoc Touch imaging system (Bio-Rad), and data were analysed using Image Lab software version 5.2 (Bio-Rad). The antibodies used were MAF bZIP transcription factor A (MafA) (rabbit, Abcam; catalogue number ab264418, RRID: AB_3096425, 1:1000 dilution), GAPDH (rabbit, Cell Signaling Technology; catalogue number, RRID: AB_10622025, 1:5000 dilution) and anti-rabbit IgG HRP (goat, Daka Cytomation, 1:5000 dilution).

### Insulin content

Insulin content was measured using a human insulin ELISA kit (Mercodia) and normalised to DNA content, which was assessed using a Quant-iT PicoGreen dsDNA assay kit (Thermo Fisher Scientific).

### Statistical analysis

Statistical analysis was performed using GraphPad Prism version 10 and Python (version 3.8.5). Two-tailed paired Student’s *t* tests, Wilcoxon rank sum tests or one-way ANOVA with correction for multiple testing were used as stated in the figure legends. Group mean differences in gene expression between cells cultured under different conditions were compared using the ΔΔ*C*_t_ method with β-actin as the reference gene. Masking was not carried out for experiments. Values shown are means ± SEM, and tests were considered significant when *p*<0.05.

## Results

### *BMP5* is heterogeneously expressed in pancreatic beta cells and is associated with increased maturity and metabolic activity

We first evaluated the expression of *BMP5* in primary human beta cells using a publicly available scRNA-seq dataset [29]. The ND (non-diabetic) dataset comprises 17,671 cells from 27 donors. We observed that *BMP5* was heterogeneously expressed in the beta cell cluster (Fig. 1a, b). This was confirmed by a CV analysis showing a CV of 212% for *BMP5*, compared with 7.8% and 29.8% for *INS* and *GAPDH*, respectively. We then categorised the beta cells into BMP5^high^ cells (4496 cells) and BMP5^low^ cells (13,175 cells) based on normalised gene expression.

Next, we assessed whether beta cell identity was affected in these two subpopulations. We defined a beta cell identity ‘score’ based on the signature identified by van Gurp et al (a list of 127 beta cell-specific genes) [31]. We found that the BMP5^high^ beta cells had a higher beta cell identity score compared with BMP5^low^ beta cells (Fig. 1c, *p*<0.001). In line with this result, expression of the beta cell maturity markers *MAFA*, *PDX1* and *NKX6.1* was significantly higher in BMP5^high^ beta cells than in BMP5^low^ beta cells (Fig. 1d, *p*<0.001).

A pathway analysis revealed that BMP5^high^ beta cells displayed upregulation of genes associated with transcription (gene expression), translation, protein trafficking and protein synthesis (Fig. 1e), indicating that BMP5^high^ beta cells were more metabolically active than BMP5^low^ beta cells. Together, these data indicate that *BMP5* expression in primary human beta cells is correlated with enhanced beta cell maturity and activity.

### *BMP5* expression is upregulated in beta cells derived from donors with type 2 diabetes

**Fig. 2:**
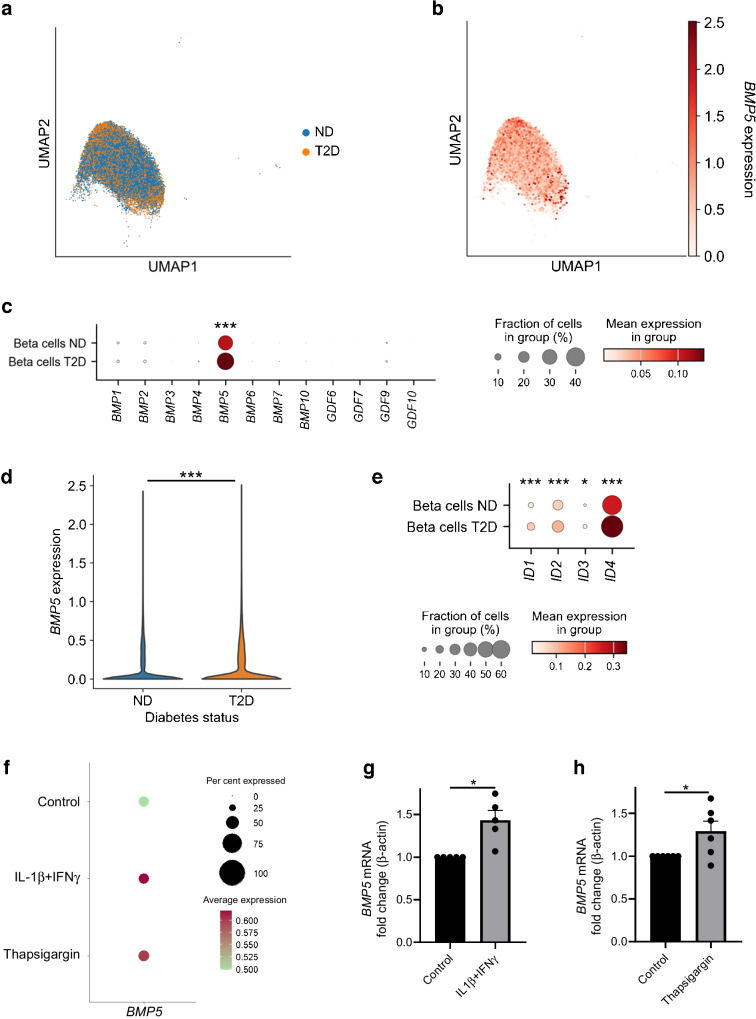
(**a**–**e**) Publicly available dataset from the Human Pancreas Analysis Program. (**a**) Uniform manifold approximation and projection (UMAP) of beta cells derived from donors without diabetes (ND) and donors with type 2 diabetes (T2D) (ND: 27 donors, 17,671 beta cells; T2D: 17 donors, 7344 beta cells). (**b**) UMAP with *BMP5* expression in beta cells derived from donors without diabetes and donors with type 2 diabetes. (**c**) Dot plot of *BMP* ligand expression in beta cells from donors without diabetes (ND) and beta cells from donors with type 2 diabetes (T2D). (**d**) Violin plot of *BMP5* expression in beta cells from donors with type 2 diabetes (T2D) vs beta cells from donors without diabetes (ND). (**e**) Dot plot of expression of the BMP target genes *ID1*, *ID2*, *ID3* and *ID4* in beta cells derived from donors with type 2 diabetes (T2D) vs beta cells from donors without diabetes (ND). (**f**) Our scRNA-seq dataset of primary human islets untreated or treated with beta cell stressors. Dot plot of *BMP5* gene expression in pancreatic beta cells from donors without diabetes treated with IL-1β + IFNγ or thapsigargin, compared with the untreated control (expression in the beta cell fraction). (**g**) *BMP5* expression after 72 h treatment with IL-1β + IFNγ (1 ng/ml and 50 ng/ml, respectively), compared with the untreated control (*n*=5). (**h**) *BMP5* expression in islets treated with 1 μmol/l thapsigargin for 5 h (read-outs performed after 24 h), compared with the untreated control (*n*=6). The Wilcoxon rank sum test (**c**–**f**) and paired Student’s *t* test (**g**, **h**) were used to assess statistical significance: ***p<0.001, *p<0.05

Next, we assessed the expression of *BMP5* in beta cells from donors with type 2 diabetes (17 donors, 7344 cells) or non-diabetic control donors (Fig. 2a, b) using the publicly available scRNA-seq dataset. Strikingly, *BMP5* was the only member of the BMP gene family to be expressed in beta cells (Fig. 2c). Furthermore, *BMP5* expression was significantly higher in beta cells derived from donors with type 2 diabetes than in beta cells from donors without diabetes (1.2-fold, *p*<0.001, Fig. 2d). This effect was associated with increased BMP/SMAD signalling in beta cells from donors with type 2 diabetes, as assessed by upregulated expression of the BMP target genes *ID1*, *ID2*, *ID3* and *ID4* (1.8-, 1.4-, 2.2- and 1.4-fold, respectively, *p*<0.05, Fig. 2e).

We then determined *BMP5* expression in our scRNA-seq dataset of primary human islets (from non-diabetic donors) untreated or treated with beta cell stressors associated with the development of type 1 and type 2 diabetes. We found a trend towards upregulation of *BMP5* in beta cells exposed to proinflammatory cytokines (IL-1β + IFNγ, results at 24 and 72 h combined, *n*=3 donors) or to an ER stress inducer (thapsigargin, 24 and 72 h combined, *n*=3 donors), compared with untreated beta cells (Fig. 2f, not significant). This trend was further validated experimentally in primary human islets. Treatment with IL-1β + IFNγ or with thapsigargin induced upregulation of *BMP5* as assessed by quantitative PCR (72 h IL-1β + IFNγ: 1.4-fold, *p*<0.05, *n*=5, Fig. 2g; 24 h thapsigargin: 1.3-fold, *p*<0.05, *n*=6, Fig. 2h). Taken together, these data show that BMP5 signalling is enhanced in beta cells in type 2 diabetes and upon ER stress and inflammation.

### BMP5 activates a cellular stress response in beta cells

**Fig. 3:**
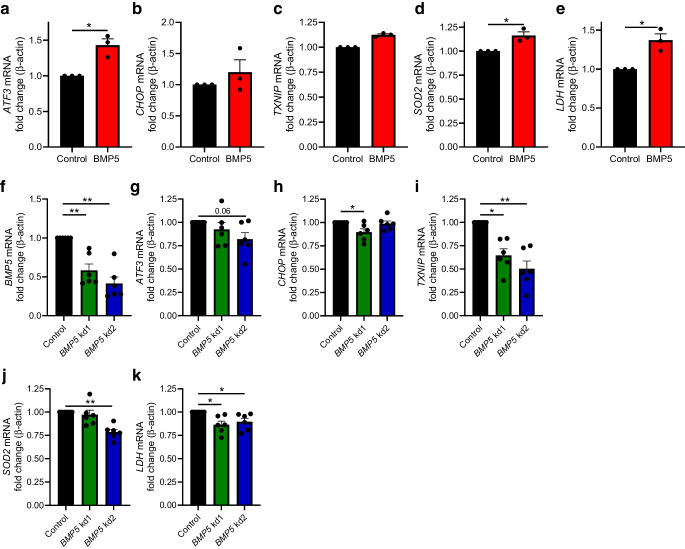
(**a**–**c**) Expression of ER stress marker genes in EndoC-βH1 cells untreated or treated with 50 ng/ml BMP5 (*n*=3): (**a**) *ATF3*, (**b**) *CHOP*, (**c**) *TXNIP*. (**d**, **e**) Expression of genes related to mitochondrial activity in EndoC-βH1 cells treated with BMP5 or untreated (*n*=3): (**d**) *SOD2*, (**e**) *LDH*. (**f**) *BMP5* expression in EndoC-βH1 cells treated with lentiviral constructs against the *BMP5* gene or control (*n*=6). (**g**–**i**) Expression of (**g**) *ATF3*, (**h**) *CHOP*, (**i**) *TXNIP* in *BMP5* knockdown or control EndoC-βH1 cells (*n*=6). (**j**, **k**) Expression of (**j**) *SOD2* and (**k**) *LDH* in *BMP5* knockdown or control EndoC-βH1 cells (*n*=6). A paired Student’s *t* test (**a**–**e**) or one-way ANOVA with correction for multiple testing (**f**–**k**) was used to assess statistical significance: **p<0.01, *p<0.05; *p*=0.06 in (**g**)

To determine whether enhanced BMP5 signalling is causing cellular stress in human beta cells, and given that primary human islets are a limited resource, we chose to perform some studies in the well-characterised and widely used human beta cell line EndoC-βH1 [38]. We exposed cells to recombinant BMP5 for 72 h, and evaluated the effect of the treatment on expression of ER stress genes and genes related to mitochondrial activity. We found that recombinant BMP5 increased *ATF3* expression 1.4-fold (Fig. 3a, *p*<0.05), while expression of the other unfolded protein response-related genes *CHOP* (also known as *DDIT3*) and *TXNIP* was not significantly altered (Fig. 3b, c). Further, expression of the mitochondrial-related genes *SOD2* and *LDH* (also known as *LDHA*) was upregulated upon BMP5 treatment (1.2- and 1.4-fold, respectively, *p*<0.05, Fig. 3d, e).

We also investigated the effect of *BMP5* downregulation using lentivirus-mediated shRNA expression. Two shRNAs were used (kd1 and kd2). *BMP5* expression was reduced by 42% and 59%, compared with the control, for kd1 and kd2, respectively (Fig. 3f, *p*<0.01). The knockdown of *BMP5* modestly reduced *CHOP* and *SOD2* expression in EndoC-βH1 cells treated with one of the *BMP5* knockdown constructs compared with the control condition (10% and 21% reduction for *CHOP* expression when using the *BMP5* kd1 construct and *SOD2* expression when using the *BMP5* kd2 construct, respectively; *p*<0.05) (Fig. 3h, j). The two knockdown constructs reduced *LDH* expression and strongly reduced *TXNIP* gene expression compared with the control (14% and 10% reductions for *LDH* when using *BMP5* kd1 and *BMP5* kd2, respectively, and 35% and 50% reductions for *TXNIP* when using *BMP5* kd1 and *BMP5* kd2, respectively; *p*<0.05) (Fig. 3i, k).

Overall, enhanced BMP5 signalling in beta cells was found to increase expression of mitochondrial-related activity genes while downregulation of *BMP5* had the opposite effect.

### Enhanced BMP5 signalling reduces glucose-stimulated insulin secretion and respiration

**Fig. 4:**
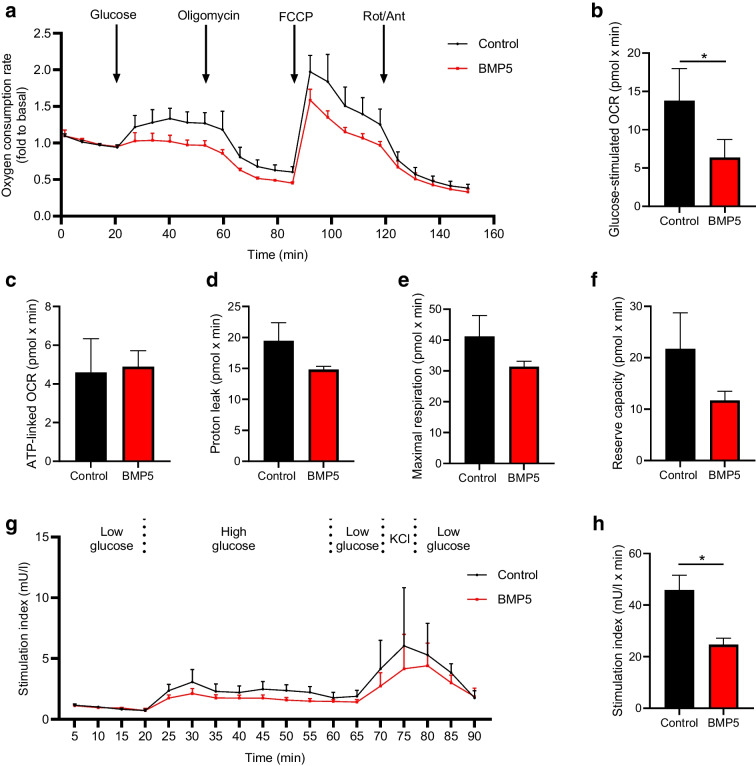
(**a**) OCR in primary human islets untreated or treated with 50 ng/ml BMP5 (72 h, *n*=4). FCCP, carbonyl cyanide-*p*-trifluoromethoxyphenylhydrazone; Rot/Ant, rotenone/antimycin A. (**b**–**f**) Bar graphs for (**b**) glucose-stimulated OCR, (**c**) ATP-linked respiration, (**d**) proton leak, (**e**) maximal respiration, and (**f**) reserve capacity in BMP5-treated islets compared with untreated control islets (*n*=4). (**g**) GSIS in primary human islets untreated or treated with 50 ng/ml BMP5 for 72 h (*n*=3). (**h**) Bar graph of insulin stimulation index of islets treated with BMP5 compared with untreated control (*n*=3). Area under the curve calculations for data shown in (**g**). A paired Student’s *t* test was used to assess statistical significance: *p<0.05

Our findings so far indicate a role for BMP5 in regulating mitochondrial activity. To further explore the influence of BMP5 on mitochondrial function, the mitochondrial OCR was examined in primary human islets exposed to recombinant BMP5. BMP5 treatment reduced the OCR upon glucose stimulation compared with the untreated control (0.5-fold, *p*<0.05, Fig. 4a, b). No significant changes in ATP-linked OCR, proton leak, maximal respiration or reserve capacity were observed in islets exposed to BMP5 (Fig. 4c–f).

Furthermore, as mitochondrial respiration upon glucose stimulation is closely linked to insulin secretion, we evaluated the effect of BMP5 treatment on beta cell function. We found that enhanced BMP5 signalling reduced glucose-stimulated insulin secretion (GSIS) in human islets compared with the control condition (0.5-fold, *p*<0.05, Fig. 4g, h), with no effect on cell death and insulin content (ESM Fig. 1b, c). Collectively, enhanced BMP5 signalling reduced glucose-stimulated oxygen consumption and insulin secretion in human islets.

### Downregulation of *BMP5* enhances beta cell function and expression of beta cell maturity markers

**Fig. 5:**
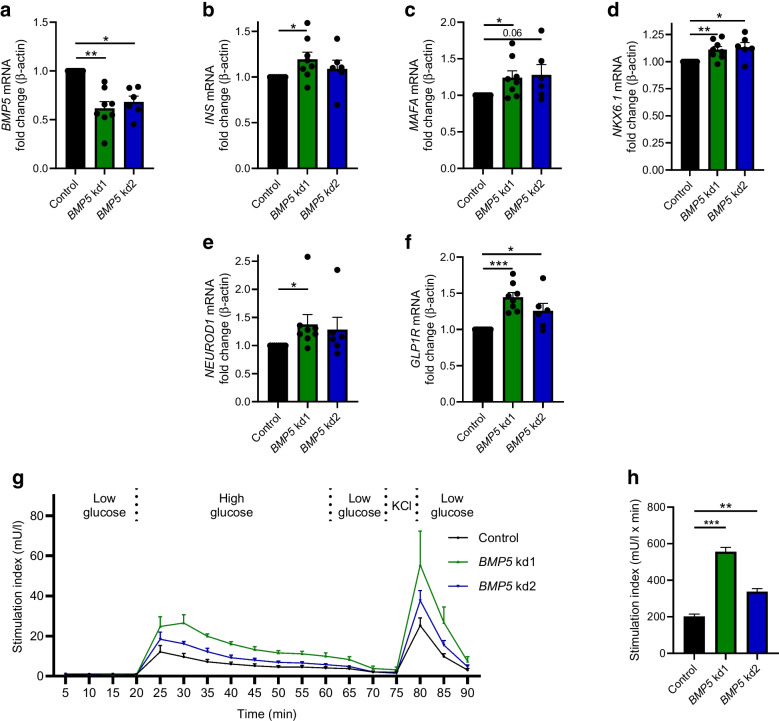
(**a**) *BMP5* gene expression in primary human islets treated with lentiviral knockdown of *BMP5* compared with control islets (control: *n*=8; *BMP5* kd1: *n*=8; *BMP5* kd2: *n*=6). (**b**–**f**) Expression of (**b**) *INS*, (**c**) *MAFA*, (**d**) *NKX6.1*, (**e**) *NEUROD1* and (**f**) *GLP1R* in islets knocked down for *BMP5* expression or control islets (control: *n*=8; *BMP5* kd1: *n*=8; *BMP5* kd2: *n*=6). (**g**) GSIS in primary human islets treated with control shRNA or lentiviral constructs against *BMP5* (*n*=3). (**h**) Bar graph of insulin stimulation index of islets treated by knockdown of *BMP5* compared with control islets (*n*=3). Area under the curve calculations for data shown in (**g**). One-way ANOVA with correction for multiple testing was used to assess statistical significance: ***p<0.001, **p<0.01, *p<0.05; *p*=0.06 in (**c**)

Finally, we investigated whether downregulation of *BMP5* expression would alter beta cell function. We transduced primary human islets with lentiviral vectors expressing shRNA against *BMP5*, leading to 38% and 32% reductions in *BMP5* expression compared with the non-target control (kd1 and kd2, respectively, *p*<0.05, Fig. 5a).

Interestingly, *INS* and *MAFA* (which encodes MafA, a master regulator of beta cell function) were upregulated by 19% and 24%, respectively, compared with control, upon *BMP5* knockdown (kd1) (Fig. 5b, c, *p*<0.05). A trend towards increased MafA expression was also observed at the protein level (ESM Fig. 1d, e; as the number of data points was only *n*=2 for this experiment we could not perform statistical analysis). Expression of other beta cell genes (*NKX6.1*, *NEUROD1* and *GLP1R*) was similarly upregulated (Fig. 5d–f, *p*<0.05; *NKX6.1*: kd1 11%, kd2 13%; *NEUROD1*: kd1 38%; *GLP1R*: kd1 44%, kd2 26%).

This effect was associated with an increase in GSIS (Fig. 5g, h, stimulation indexes of 12, 25 and 18 for control, *BMP5* kd1 and *BMP5* kd2, respectively, *p*<0.01), with no effect on insulin content (ESM Fig. 1f). The effect on increased beta cell function was seen predominantly in *BMP5* kd1 islets, as for the increase in beta cell gene expression.

All in all, these data show that BMP5 regulates insulin secretion, and reduced *BMP5* gene expression in beta cells augments beta cell function.

## Discussion

Type 2 diabetes is a complex multifactorial disease that is defined by beta cell failure and insulin resistance [39]. Given the increasing global prevalence of this disease [40], unravelling the mechanisms underlying beta cell failure is of pronounced importance. Here, we showed that enhanced levels of BMP5 signalling may contribute to beta cell failure, through decreased mitochondrial activity and insulin secretion upon high glucose.

BMP5 is the main BMP ligand expressed in primary human beta cells. It is expressed in the more mature and metabolically active beta cells. Furthermore, we showed that its expression is enhanced in beta cells from donors with type 2 diabetes, and under proinflammatory and ER stress conditions associated with beta cell failure. The apparent discrepancy with the original paper describing this dataset [29], which did not report alterations in *BMP5* expression in beta cells derived from type 2 diabetes donors, may be attributed to a difference in computational analysis. Our methods involved individual gene counts per cell, while Elgamal et al used a pseudo-bulk approach. Overall, more studies, beyond single-cell transcriptomics, are needed to investigate further the potential causality of altered *BMP5* expression in the development of beta cell failure in type 2 diabetes.

In this study, increased BMP5 signalling induces expression of mitochondrial-related genes, and this effect is associated with reduced mitochondrial oxygen consumption and reduced insulin secretion upon glucose stimulation. In contrast, we observed a reduction of mitochondrial-related genes upon downregulation of *BMP5* expression. In line with our data, a study performed in adipose-derived stem cells indicated reduced expression of mitochondrial genes after *BMP5* silencing [28]. The authors also observed reduced reactive oxygen species neutralisation capacity, mitochondrial membrane potential and ATP production. On the other hand, treatment with recombinant BMP5 was able to maintain mitochondrial membrane potential, and increased ATP production in high glucose-treated Schwann cells.

Glucose-stimulated mitochondrial respiration is one of the key features of a functional islet, and is highly correlated with GSIS [41–44]. In addition, we show here that reduced levels of *BMP5* result in increased beta cell function. Collectively, these findings show that alteration of *BMP5* expression in beta cells has implications for beta cell function, highlighting the relevance of the function of this novel beta cell marker.

Downregulation of *BMP5* gene expression in primary human islets, although not confirmed at the protein level due to technical challenges, induced upregulation of *INS*, *NKX6.1*, *NEUROD1* and *GLP1R*, as well as *MAFA*. MafA has been described as the master regulator of insulin secretion [45–47]. We and others have shown that its expression is reduced in type 2 diabetes as well under stress conditions that are associated with reduced beta cell function [47–49]. Overexpression of *MAFA* in mice led to an increase in expression of the *INS* and *GLP1R* genes, and enhanced GSIS [45]. In accordance with the effect of MafA on beta cells, NKX6.1 has been reported to be essential for preserving the identity of pancreatic beta cells, and it controls GSIS and the expression of genes responsible for insulin processing [50–52].

We showed that recombinant treatment of BMP5 reduced GSIS. This is in line with the effect of other BMP ligands, BMP2 and BMP4, that have been shown to impair GSIS [53–56]. A potential mechanism has been recently proposed by Urizar et al, in which BMP2 induces loss of beta cell maturity and functionality through histone modification, leading to the reduced binding of neurogenic differentiation 1 (NeuroD1) to the promoters of its target genes, which include the beta cell markers *MAFA*, *GLP1R* and *INS* [54]. A similar mechanism may be involved in BMP5 regulation of beta cell identity and function.

We found that *BMP5* expression is upregulated under proinflammatory and ER stress conditions. Other BMP ligands have been demonstrated to contribute to the detrimental effects of inflammation, such as in the pathophysiology of vascular endothelial cells [57, 58] and synoviocytes [59, 60], as well as in rat beta cells, mouse islets and human islets [53, 54, 61]. A single-cell multi-omics study showed that expression of the *BMP5* gene and those encoding its receptors (*BMPR2*, *BMPR1A*, *ACVR1*, *ACVR2A* and *ACVR2B*) was significantly upregulated in stressed beta cells (characterised by elevated expression of *NPTX2* and *GDF15*) [62].

Enhanced *BMP5* expression in beta cells may reflect a protective response to stress. For instance, BMP5 has been shown to promote the survival of various cell types, including cardiomyocytes after myocardial infarction [63], neural crest progenitor cells [64] and sciatic nerve cells [28]. However, *BMP5* downregulation was reported to decrease chondrocyte apoptosis in an osteoarthritis disease model [65], reflecting a context-dependent role for BMP5 in cell survival. Furthermore, we observed a marked decrease in *TXNIP* gene expression in beta cells knocked down for expression of the *BMP5* gene. Thioredoxin-interacting protein (TXNIP) is an important regulator of beta cell apoptosis [66]. Depletion of TXNIP has been shown to enhance beta cell survival through increased Akt/Bcl-xL signalling, and TXNIP deficiency augments insulin secretion [67, 68]. Whether BMP5 contributes to a protective response to stress in the context of diabetes remains to be further investigated.

To conclude, we showed that *BMP5* is the predominant BMP family gene member in human beta cells, and that changes in *BMP5* expression are associated with altered beta cell function. BMP5 (or its downstream pathway) may represent a novel target for preservation and/or recovery of beta cell function, as currently being evaluated for other medical conditions [69].

## Supplementary Information

Below is the link to the electronic supplementary material.

ESM (PDF 233 KB)

## Data Availability

The data that support the findings of this study are available from the corresponding author upon reasonable request.

## Abbreviations

ATF3: Activating transcription factor 3
BMP: Bone morphogenetic protein
CHOP: C/EBP homologous protein
GSIS: Glucose-stimulated insulin secretion
kd1, kd2: Knockdown constructs
MafA: MAF bZIP transcription factor A
OCR: Oxygen consumption rate
scRNA-seq: Single-cell RNA sequencing
shRNA: Short hairpin RNA
TXNIP: Thioredoxin-interacting protein
