# Supplementary material for: BMP5 signalling in beta cells and the impact on insulin secretion in the context of type 2 diabetes
Source: Diabetologia. 2025 Jun 5;68(9):1983–96. doi: 10.1007/s00125-025-06457-9 (PMC12361344; doi:10.1007/s00125-025-06457-9)
Supplement: Supplementary file 1 — ESM (PDF 233 KB) [file 125_2025_6457_MOESM1_ESM.pdf]

ESM Table 1: Donor characteristics

Checklist for reporting human islet preparations used in research

Adapted from Hart NJ, Powers AC (2018) Progress, challenges, and suggestions for using human islets to understand islet biology and human diabetes. Diabetologia https://doi.org/10.1007/s00125-018-4772-2

**Islet preparation**	**1**	**2**	**3**	**4**	**5**	**6**	**7**
**MANDATORY INFORMATION**							
Unique identifier	P652	R189	P655	R186	R190	R155	R161
Donor age (years)	49	78	46	70	70	62	48
Donor sex (M/F)	M	F	F	M	M	M	F
Donor BMI (kg/m^2)	26	21	32	25	24	24	33
Donor HbA_{1c} or other measure of blood glucose control	5.4	5.9		5.6	5.6		5.7
Origin/source of islets[b]	LUMC	LUMC	LUMC	LUMC	LUMC	LUMC	LUMC
Islet isolation centre	LUMC	LUMC	LUMC	LUMC	LUMC	LUMC	LUMC
Donor history of diabetes? Please select yes/no from drop down list	No	No	No	No	No	No	No
**If Yes, complete the next two lines if this information is available**							
Diabetes duration (years)							
Glucose-lowering therapy at time of death[c]							
**RECOMMENDED INFORMATION**							
Donor cause of death	Trauma: Capitis	Sub Arachnoidal Bleeding	Intra Cerebral Bleeding	Sub Arachnoidal Bleeding	Suicide	Sub Arachnoidal Bleeding	Circulational: Cardiac Arrest
Warm ischaemia time (h)							
Cold ischaemia time (h)							
Estimated purity (%)							
Estimated viability (%)							
Total culture time (h)[d]							
Glucose-stimulated insulin secretion or other functional measurement[e]							
Handpicked to purity? Please select yes/no from drop down list							
Additional notes							

Islet preparation	8	9	10	11	12	13
**MANDATORY INFORMATION**						
Unique identifier	R163	R165	R167	R171	R249	R251
Donor age (years)	55	40	56	72	70	54
Donor sex (M/F)	F	M	M	M	M	M
Donor BMI (kg/m^2)	22	19	24	19	27	24
Donor HbA_{1c} or other measure of blood glucose control	6.4	5.5			5.6	
Origin/source of islets[b]	LUMC	LUMC	LUMC	LUMC	LUMC	LUMC
Islet isolation centre	LUMC	LUMC	LUMC	LUMC	LUMC	LUMC
Donor history of diabetes? Please select yes/no from drop down list	No	No	No	No	No	No
Diabetes duration (years)						
**If Yes, complete the next two lines if this information is available**						
Glucose-lowering therapy at time of death[c]						
**RECOMMENDED INFORMATION**						
Donor cause of death	Circulational: Cardiac Arrest	Circulational: Cardiac Arrest	Sub Arachnoidal Bleeding	Circulational: Cardiac Arrest	Cerebral Ischemia	Respirational
Warm ischaemia time (h)						
Cold ischaemia time (h)						
Estimated purity (%)						
Estimated viability (%)						
Total culture time (h)[d]						
Glucose-stimulated insulin secretion or other functional measurement[e]						
Handpicked to purity? Please select yes/no from drop down list						
Additional notes						

**Islet preparation**	**14**	**15**	**16**	**17**	**18**	**19**
**MANDATORY INFORMATION**						
Unique identifier	R254	R255	P721	R244	R245	R260
Donor age (years)	61	66	46	72	69	51
Donor sex (M/F)	M	F	M	F	M	F
Donor BMI (kg/m^2)	24	31	30	27	29	20
Donor HbA_{1c} or other measure of blood glucose control		6.1	5.9	5.3	5.2	5.8
Origin/source of islets[b]	LUMC	LUMC	LUMC	LUMC	LUMC	LUMC
Islet isolation centre	LUMC	LUMC	LUMC	LUMC	LUMC	LUMC
Donor history of diabetes? Please select yes/no from drop down list	No	No	No	No	No	No
**If Yes, complete the next two lines if this information is available**						
Diabetes duration (years)						
Glucose-lowering therapy at time of death[c]						
**RECOMMENDED INFORMATION**						
Donor cause of death	Sub Arachnoidal Bleeding	Sub Arachnoidal Bleeding	Circulational	Circulational	Circulational: Cardiac Arrest	Intra Cerebral Bleeding
Warm ischaemia time (h)						
Cold ischaemia time (h)						
Estimated purity (%)						
Estimated viability (%)						
Total culture time (h)[d]						
Glucose-stimulated insulin secretion or other functional measurement[e]						
Handpicked to purity? Please select yes/no from drop down list						
Additional notes						

**Islet preparation**	**20**	**21**	**22**	**23**	**24**	**25**
**MANDATORY INFORMATION**						
Unique identifier	R261	P761	P762	P765	R268	P775
Donor age (years)	55	47	56	73	22	27
Donor sex (M/F)	M	M	M	F	F	M
Donor BMI (kg/m^2)	28	26	32	26	30	22
Donor HbA_{1c} or other measure of blood glucose control	6	5.5	5.9	5.3		5
Origin/source of islets[b]	LUMC	LUMC	LUMC	LUMC	LUMC	LUMC
Islet isolation centre	LUMC	LUMC	LUMC	LUMC	LUMC	LUMC
Donor history of diabetes? Please select yes/no from drop down list	No	No	No	No	No	No
**If Yes, complete the next two lines if this information is available**						
Diabetes duration (years)						
Glucose-lowering therapy at time of death[c]						
Donor cause of death	Suicide	Circulational: Cardiac Arrest	Trauma: Capitis	Intra Cerebral Bleeding	Respirational	Euthanasia
Warm ischaemia time (h)						
Cold ischaemia time (h)						
Estimated purity (%)						
Estimated viability (%)						
Total culture time (h)[d]						
Glucose-stimulated insulin secretion or other functional measurement[e]						
Handpicked to purity? Please select yes/no from drop down list						
Additional notes						

ESM Table 2: List of human primers

Gene name	Sequence primer forward (5'→3')	Sequence primer reverse (5'→3')
ACTIN	TGCGTGACATTAAGGAGAAG	TGAAGGTAGTTTCGTGGATG
ATF3	GTGCCGAAACAAGAAGAAGG	TCTGAGCCTTCAGTTCAGCA
BMP5	AAGAGGACAAGAAGGACTAAAAATAT	GTAGAGATCCAGCATAAAGAGAGGT
CHOP	GACCTGCAAGAGGTCCTGTC	CTCCTCCTCAGTCAGCCAAG
GLP1R	GACGCTCAAGAATCCTCTGG	TCTCAAGAGACAGCGTGTGG
INS	AAGAGGCCATCAAGCAGATCA	CAGGAGGCGCATCCACA
LDH	TGGCAGCCTTTTCCTTAGAA	CGCTTCCAATAACACGGTTT
MAFA	CAGTCCTGCCGCTTCAAG	ACAGGTCCCGCTCTTTGG
NEUROD1	TGAGACTATCACTGCTCAGG	CACTCTCGCTGTACGATTTG
NKX6.1	CTGGCCTGTACCCCTCATCA	CTTCCCGTCTTTGTCCAACAA
SOD2	GGAAGCCATCAAACGTGACT	CTGATTTGGACAAGCAGCAA
TXNIP	GGTCTTTAACGACCCTGAAAAGG	ACACGAGTAACTTCACACACCT

ESM Figure 1

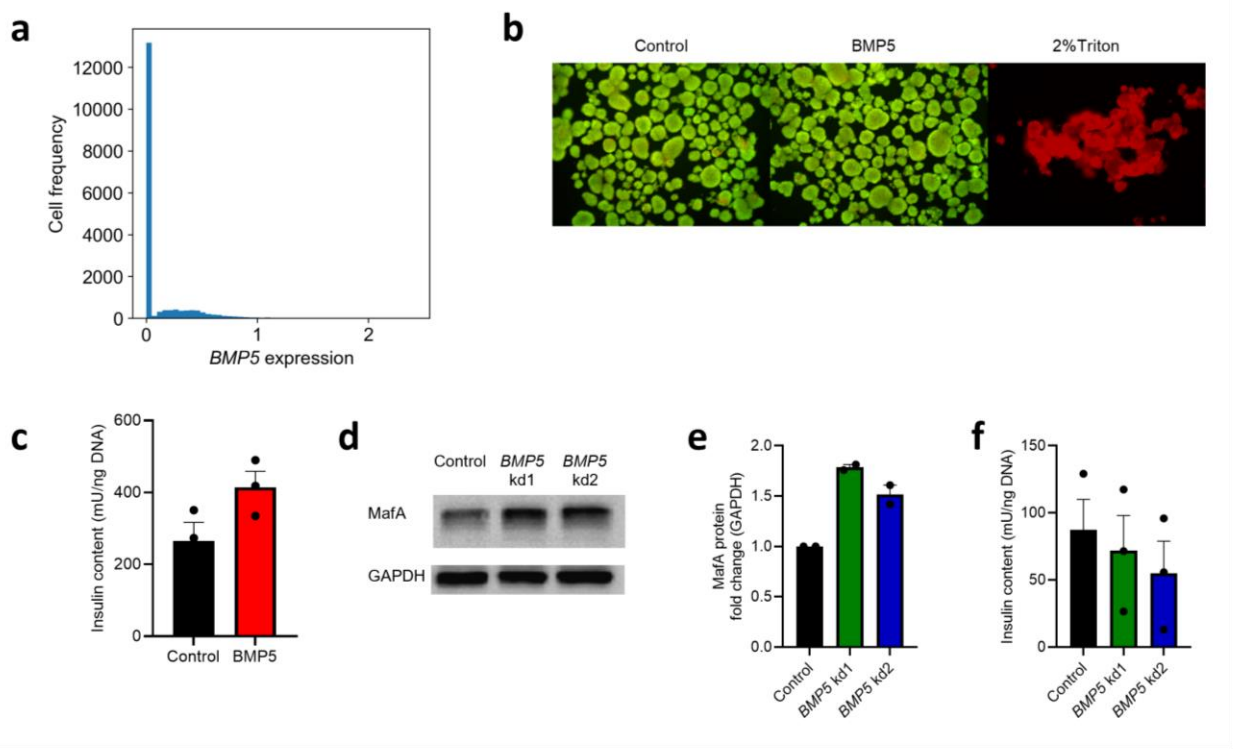

ESM Fig. 1 (**a**) Cut off $BMP5^{low}$ and $BMP5^{high}$ assigned beta cells. In $BMP5^{low}$ beta cells the expression level of *BMP5* is 0. In $BMP5^{high}$ beta cells the expression level of *BMP5* is greater than 0. (**b**) Fluorescein Diacetate (viable, green) and Propidium Iodide (dead, red) staining of primary human islets untreated or treated for 72h with BMP5 (50ng/ml) or 5 minutes with 2% Triton. (**c**) Insulin content of primary human islets treated with recombinant BMP5 (50ng/ml) or untreated for 72h. (**d**) Representative Western Blot of MafA protein expression in human islets treated with constructs against the *BMP5* gene or control. (**e**) Bar graph of MafA protein expression in human islets treated with constructs against the *BMP5* gene or control (n=2). (**f**) Insulin content of primary human islets treated with constructs against the *BMP5* gene or control.
